# Supplementary material for: Flexing the principal gradient of the cerebral cortex to suit changing semantic task demands
Source: eLife. 2022 Sep 28;11:e80368. doi: 10.7554/eLife.80368 (PMC9555860; doi:10.7554/eLife.80368)
Supplement: Supplementary file 1. [file elife-80368-supp1.docx]

**Supplementary Materials.**

Table S1.

Spin correlation between gradients estimated by traditional connectivity using resting state data and informational connectivity using semantic and chevron neural responses in each hemisphere.

|  |  | Semantic Task | | | | | |
| --- | --- | --- | --- | --- | --- | --- | --- |
|  |  | Left Hemisphere | | | Right Hemisphere | | |
|  |  | Gradient 1 | Gradient 2 | Gradient 3 | Gradient 1 | Gradient 2 | Gradient 3 |
| Resting | Gradient 1 | 0.867*** |  |  | 0.874*** |  |  |
|  | Gradient 2 |  | 0.421*** |  |  | 0.439*** |  |
|  | Gradient 3 |  |  | 0.419*** |  |  | -0.708*** |
|  |  | Chevron Task | | | | | |
|  |  | Left Hemisphere | | | Right Hemisphere | | |
|  |  | Gradient 1 | Gradient 2 | Gradient 3 | Gradient 1 | Gradient 2 | Gradient 3 |
| Resting | Gradient 1 | 0.803*** |  |  | 0.806*** |  |  |
|  | Gradient 2 |  | 0.261*** |  |  | 0.246*** |  |
|  | Gradient 3 |  |  | -0.060*** |  |  | -0.833*** |

Gradients 1 and 2 showed similar spatial patterns when derived from informational connectivity metrics in the semantic and chevron tasks and from resting state data. However, gradient 3 showed a task-specific state which captured the separation of left hemisphere from right hemisphere for the semantic task.
