# Supplementary material for: Flexing the principal gradient of the cerebral cortex to suit changing semantic task demands
Source: eLife. 2022 Sep 28;11:e80368. doi: 10.7554/eLife.80368 (PMC9555860; doi:10.7554/eLife.80368)
Supplement: Transparent reporting form [file elife-80368-transrepform1.docx]

***eLife’s* transparent reporting form**

We encourage authors to provide detailed information *within their submission* to facilitate the interpretation and replication of experiments. Authors can upload supporting documentation to indicate the use of appropriate reporting guidelines for health-related research (see [EQUATOR Network](http://www.equator-network.org/%20)), life science research (see the [BioSharing Information Resource](https://biosharing.org/)), or the [ARRIVE guidelines](http://www.plosbiology.org/article/info:doi/10.1371/journal.pbio.1000412) for reporting work involving animal research. Where applicable, authors should refer to any relevant reporting standards documents in this form.

If you have any questions, please consult our Journal Policies and/or contact us: [editorial@elifesciences.org](mailto:editorial@elifesciences.org).

**Sample-size estimation**

- You should state whether an appropriate sample size was computed when the study was being designed
- You should state the statistical method of sample size computation and any required assumptions
- If no explicit power analysis was used, you should describe how you decided what sample (replicate) size (number) to use

Please outline where this information can be found within the submission (e.g., sections or figure legends), or explain why this information doesn’t apply to your submission:

No explicit power analysis was performed to determine the sample size in this experiment, however, the present sample sizes for task-based fMRI data analysis (N=31) is comparable to, or significantly exceeds, that of prior task-based fMRI studies examining the neural basis of semantic cognition (e.g., Ubaldi et al., 2022, J.Neuroscience; Gao et al., 2022, Cerebral Cortex; Sormaz et al., 2018, PNAS).

**Replicates**

- You should report how often each experiment was performed
- You should include a definition of biological versus technical replication
- The data obtained should be provided and sufficient information should be provided to indicate the number of independent biological and/or technical replicates
- If you encountered any outliers, you should describe how these were handled
- Criteria for exclusion/inclusion of data should be clearly stated
- High-throughput sequence data should be uploaded before submission, with a private link for reviewers provided (these are available from both GEO and ArrayExpress)

Please outline where this information can be found within the submission (e.g., sections or figure legends), or explain why this information doesn’t apply to your submission:

1. In the experiment, each trial began with a visually presented word-pair for 4.5s, followed by 1.5s in which participants rated the strength of the link they retrieved. Semantic trials were separated by an easy chevron task for 6s: participants pressed buttons to indicate whether each chevron faced left or right, with 10 chevrons presented. We included 144 trials in total and collected neuroimaging/behavioral data from 36 participants (5 participants were excluded in the final analysis). This task is detailed described in the ***Methods*** section, and is visualized in Figure 1.

2. The details of exclusion/inclusion of data can be found in ***Participant****s* section: Five participants were not included in the data analysis: one participant had poor behavioural performance (no link made on 32% of trials), another withdrew during scanning, one scan showed a structural anomaly, one had missing volumes and another showed excessive head movement (movement > 1.55mm).

3. For the potential outliers in the brain data, due to the lower tSNR in limbic network, we conducted additional control analysis to examine whether our results on dimensionality was robust after excluding areas in this network. The details of how the outliers were handled can be found in ***Results*** (Representational space changed along the principal gradient) section.

4. We have uploaded our Neuroimaging data at the group-level in Neurovault, this information can be found in Data availability section: Neuroimaging data at the group level are openly available in Neurovault at https://neurovault.org/collections/12539/.

Other information is not applicable to the fMRI analyses performed in this study.

**Statistical reporting**

- Statistical analysis methods should be described and justified
- Raw data should be presented in figures whenever informative to do so (typically when N per group is less than 10)
- For each experiment, you should identify the statistical tests used, exact values of N, definitions of center, methods of multiple test correction, and dispersion and precision measures (e.g., mean, median, SD, SEM, confidence intervals; and, for the major substantive results, a measure of effect size (e.g., Pearson's r, Cohen's d)
- Report exact p-values wherever possible alongside the summary statistics and 95% confidence intervals. These should be reported for all key questions and not only when the p-value is less than 0.05.

Please outline where this information can be found within the submission (e.g., sections or figure legends), or explain why this information doesn’t apply to your submission:

1. The statistical tests used were reported using text and we reported statistics with degrees of freedom when applicable. Results were reported using mean ± SEM (e.g., in Figure 2 and Figure 3) and exact *p*-values when applicable (e.g., behavioral results in Experiment 1).

2. For the statistical correlation analysis between gradient and dimensionality and semantic-brain alignment, we reported exact coefficient r-values and p-values. Bonferroni correction was also applied in dimensionality/gradient difference in functional networks, to account for the fact that we included four brain networks, the *p*-value consequently accepted as significant was *p* < 0.0125.

3. FDR correction was applied to define significant cluster using q = 0.05 in dimensionality difference and semantic-brain alignment analysis (e.g., in Figure 3 and Figure 4).

All statistical analysis methods are described in the ‘Results’ and ‘Methods’ sections.

(For large datasets, or papers with a very large number of statistical tests, you may upload a single table file with tests, Ns, etc., with reference to sections in the manuscript.)

**Group allocation**

- Indicate how samples were allocated into experimental groups (in the case of clinical studies, please specify allocation to treatment method); if randomization was used, please also state if restricted randomization was applied
- Indicate if masking was used during group allocation, data collection and/or data analysis

Please outline where this information can be found within the submission (e.g., sections or figure legends), or explain why this information doesn’t apply to your submission:

This study examines the underlying neurocognitive mechanism of semantic cognition while conducting a semantic task using healthy participants, and therefore no group allocations were made. The details of exclusion/inclusion of participants can be found in the **Methods** section (i.e. Participants).

**Additional data files (“source data”)**

- We encourage you to upload relevant additional data files, such as numerical data that are represented as a graph in a figure, or as a summary table
- Where provided, these should be in the most useful format, and they can be uploaded as “Source data” files linked to a main figure or table
- Include model definition files including the full list of parameters used
- Include code used for data analysis (e.g., R, MatLab)
- Avoid stating that data files are “available upon request”

Please indicate the figures or tables for which source data files have been provided:

Group-level statistical maps for Figures 3-4, which can be accessed in Neurovault at <https://neurovault.org/collections/12539/>. All analysis codes, materials for semantic task, behavioral and neuroimaging data used to produce the main findings for Figure 1-4 were also available online: https://osf.io/mkgcy.

The conditions of our ethical approval do not permit public archiving of the data because participants did not provide sufficient consent. Researchers who wish to access the data should contact the Research Ethics and Governance Committee of the York Neuroimaging Centre, University of York, or the corresponding authors. Data will be released to researchers when this is possible under the terms of the GDPR (General Data Protection Regulation).
